# Ecological Characterization of the Colonic Microbiota of Normal and Diarrheic Dogs

**DOI:** 10.1155/2008/149694

**Published:** 2009-01-13

**Authors:** Julia A. Bell, Jamie J. Kopper, Judy A. Turnbull, Nicholas I. Barbu, Alice J. Murphy, Linda S. Mansfield

**Affiliations:** ^1^Comparative Enteric Diseases Laboratory, National Food Safety and Toxicology Center, Michigan State University, 181 Food Safety Building, East Lansing, MI 48824, USA; ^2^College of Veterinary Medicine, Michigan State University, G-100 Veterinary Medical Center, East Lansing, MI 48824, USA; ^3^Cell and Molecular Biology Program, Michigan State University, 2240A Biomedical and Physical Science Building, East Lansing, MI 48824, USA; ^4^Department of Microbiology and Molecular Genetics, Michigan State University, 2215 Biomedical Physical Sciences, East Lansing, MI 48824, USA

## Abstract

We used terminal restriction fragment polymorphism (T-RFLP) analysis to assess (1) stability of the fecal microbiota in dogs living in environments characterized by varying degrees of exposure to factors that might alter the microbiota and (2) changes in the microbiota associated with acute episodes of diarrhea. Results showed that the healthy canine GI tract harbors potential enteric pathogens. Dogs living in an environment providing minimal exposure to factors that might alter the microbiota had similar microbiotas; the microbiotas of dogs kept in more variable environments were more variable. Substantial changes in the microbiota occurred during diarrheic episodes, including increased levels of *Clostridium perfringens, Enterococcus faecalis*, and *Enterococcus faecium*. When diet and medications of a dog having a previously stable microbiota were changed repeatedly, the microbiota also changed repeatedly. Temporal trend analysis showed directional changes in the microbiota after perturbation, a return to the starting condition, and then fluctuating changes over time.

## 1. INTRODUCTION

Acute infectious diarrhea is a worldwide public health problem with a long list of differential diagnoses. In the US, major pathogens responsible for most cases include *Salmonella, Campylobacter, Shigella, Escherichia coli* O157:H7, and *Cryptosporidium* [1]. *Vibrio, Yersinia, Listeria, Cyclospora, Clostridium difficile, Giardia*, rotavirus, and *Entamoeba histolytica* are also reported at lower rates [1]. Enterotoxigenic, enteropathogenic, enteroaggregative, and enteroinvasive strains of *E. coli,* toxin-producing *Clostridium perfringens, Staphylococcus aureus, Bacillus cereus,* and norovirus also cause infectious diarrheas, but may not be included in routine testing [1]. Main etiologies for acute diarrhea in the dog are the same or similar to organisms seen in humans [2–6]. Other known pathogens of dogs include *Clostridium piliforme* [7], *Brachyspira* (*Serpulina*) spp. [8], *Enterococcus* spp. [9], and *Helicobacter* spp. [10–12]. In both humans and dogs, a number of bacteria, such as *Enterococcus* spp. and certain *Clostridia* spp., are recognized as opportunistic pathogens or enhance disease from other organisms when conditions are ideal for their growth and when competitors are absent [13–15]. Most cases of acute infectious diarrhea are self-limiting illnesses and resolve in a few days with or without symptomatic treatment with rehydration along with antimicrobial or antiparasitic drugs targeting the “etiological agent” [16]. The presence of known GI tract pathogens recovered or demonstrated is used to attribute an etiology during a diarrheic episode; however, causation is seldom proved.

Few animal models of functional GI disorders exist, but the dog GI tract and microbiota bear many similarities to those of humans. Dogs are monogastric omnivores in which dietary manipulations are easy to achieve and a small-sized cecum provides only a small component of hind gut fermentation. Dogs are litter bearers with a short reproductive interval, where families of related individuals are easy to acquire and where management can be manipulated to control environmental exposures. Early work has been done to describe GI microbiota in healthy dogs. Microbial community analysis of feces from 4 Labrador breed dogs was performed using culture followed by 16S rRNA gene sequencing [17]. Despite intensive efforts, these methods underestimated community diversity and skewed results toward organisms more successful on particular culture media. These results did support the use of molecular-based methodologies for determining community profiles, but at the time, sequences of many isolates were not found in the Ribosomal Database Project and EMBL databases. Suchodolski et al. described the microbial community in duodenum, jejunum, ileum, and colon contents from six healthy unrelated dogs using near-full-length 16S rRNA gene PCR and cloned libraries [18]. Here, *Firmicutes* was the most diverse and abundant phylum; *Clostridiales* was the most diverse bacterial order, forming several *Clostridium* clusters; anaerobic *Fusobacteriales* and *Bacteroidales* increased in their relative abundance along the intestinal tract, peaking in ileum and colon; and *Lactobacillales* occurred commonly in all parts of the intestine. These results on *Firmicutes* and *Clostridiales* were similar in humans [19] with *Clostridium* cluster XIVa being the predominant contributor to *Clostridiales* sequences in both dogs and humans. Furthermore, *Fusobacteria* appeared to be a minor part of the intestinal community in other species, including humans [19]. Also, *Proteobacteria*—including *E. coli*-like organisms—predominated in the duodenum and were sparse in the colon in both dogs [18] and humans [19]. In another study designed to define host distribution patterns of fecal bacteria of the order *Bacteroidales* as markers for fecal source identification in aquatic environments, human, dog, cat, and gull sequences were clustered together in phylogenetic analysis [20]. Swanson et al. performed a study using healthy dogs to examine whether prebiotic (fructooligosaccharides) or probiotic (*Lactobacillus acidophilus*) treatments would alter gut microbial populations, fermentative end products, and nutrient digestibilities [21]. In one experiment, fructooligosaccharide treatment decreased *C. perfringens* and increased fecal butyrate and lactate concentrations, while in a second experiment, this treatment increased bifidobacteria, lactobacilli, and fecal lactate and butyrate and decreased fecal ammonia, isobutyrate, isovalerate, and total branched-chain fatty acid concentrations. Finally, recent global vertebrate gut microbiota studies showed that captive (zoo) bush dogs on carnivorous diets had microbial communities that clustered with other carnivores, and that primates on omnivorous diets had fecal microbiota most like humans [22, 23]. In these studies using tree-based and network-based analyses of microbial communities, clustering by diet (herbivore, omnivore, or carnivore) was highly significant. However, these investigators did not consider the microbiota of modern pet dogs on highly processed diets and cohabiting with humans. Taken together, these studies show that dogs are a reasonable model for study of the role of microbiota in GI disorders and that understanding of the GI microbial community in dogs is at a stage of readiness for this to be pursued.

Many diarrheal diseases are attributable to specific pathogens, to polymicrobial interactions, or to shifts or imbalances in the resident microbial community in response to external stress(s). Thus, acute diarrheas can result from myriad etiologies making attribution difficult. Lately, attention has been focused on the role of the microbiota. The normal gut biota or “enterome” is a complex microbial ecosystem that plays a crucial role in maintaining GI homeostasis and in certain disease states [24]. However, 300–500 different bacterial species are estimated to inhabit the human colon, many of which are not cultivatable [25]. This estimate of diversity has changed little over the years, even with the application of molecular techniques to the study of the colonic microbiota [26, 27]. Eckburg et al. [26] used collector's curves to estimate that extensive sequencing would reveal at least 500 species. Adding to complexity during analysis are the sheer numbers of GI organisms, which can reach a density of 10^12^ organisms/gram of feces, with a total gut population of 10^14^–10^15^ microbes [28]. In the dog, breed and age were shown to have significant effects on particular aerobic and anaerobic bacterial counts using denaturing gradient gel electrophoresis of PCR amplified 16S ribosomal fragments. Here, each individual dog harbored a characteristic fecal bacterial community which was independent of diet [29]. We hypothesized that dogs have a stable composition of the colon microbial community and that episodes of diarrhea lead to long lasting changes in community composition and/or function; furthermore, treatment for specific pathogens can compound these effects. To address this hypothesis, we required (1) diarrheic perturbations of the GI tract with or without treatment to study, (2) documentation of the presence of pathogens in the GI tract, (3) a cost-effective technique for assessing shifts in the GI tract microbiota, and (4) the assurance that the microbiota in an individual is stable enough for us to be able to detect meaningful changes. We used diagnostic PCR assays to document the presence of pathogens in the GI tract. The GI tract perturbations we studied were (1) acute episodes of diarrhea with or without antibiotic treatment and (2) changes in diet and medications. The technique we chose for assessing shifts in the GI tract microbiota was terminal restriction fragment length polymorphism (T-RFLP) analysis.

T-RFLP is one of a family of related techniques used to describe microbial communities containing large numbers of organisms that are undescribed and/or difficult to cultivate. T-RFLP is based on PCR amplification and restriction enzyme digestion of 16S rDNA PCR products followed by capillary electrophoresis on a DNA sequencer. Here, data were analyzed using exploratory statistical techniques that help reveal patterns rather than the more familiar inferential statistics that help discriminate between hypotheses. Community studies using these techniques that have been reported to date have involved small numbers of samples. For example, Nielsen et al. characterized populations of lactic acid bacteria and total bacterial communities in one sample each from three colon segments in four human subjects [30]. In this study, the total communities from different parts of the colon in the same individual were similar to each other, but total communities varied between individuals. In another trial, fecal communities were studied in eight individuals of different ages and sexes; five adults and two children had similar arrays of microorganisms in their fecal communities but different proportions of the bacterial species, while a two-week-old infant had a much simpler community [31]. Thus, T-RFLP data revealed valuable results even though attribution of gains or losses of specific bacterial genera or species was not possible. Also, we recognize that many factors may affect the results obtained with the T-RFLP technique; these factors include choice of primers; choice of restriction enzymes; and various amplification biases due to PCR reaction parameters such as amount and complexity of the template DNA, annealing temperature, and number of cycles in the amplification reaction [32–35]. Nevertheless, T-RFLP is rapid, sensitive, and reproducible [31, 34], and, unlike many other community analysis techniques, it yields both taxonomic information regarding organisms in the community and estimates of their relative proportions in the total microbial population. It is considered a useful tool in the study of microbial communities and can be used to generate data that help to determine whether further studies employing more precise, laborious, and expensive techniques, such as targeted real-time PCR for the detection and quantization of specific microorganisms or the generation, sequencing, and analysis of cloned 16S rDNA libraries, are justified [31].

To address our hypothesis, we studied the microbial communities of dogs during diarrheic episodes and compared them to those of healthy control dogs to make a preliminary assessment of the contribution of members of the normal community to acute diarrheal disease processes. Results of these studies showed that fecal microbiotas varied among dogs, even those that were closely related, and were largely influenced by diet. A dog treated for diarrhea with metronidazole did exhibit loss of richness followed by return to a stable microbiota; the same treatment aimed at a second bout of diarrhea resulted in an unstable microbiota that ultimately lost richness and evenness. Thus, our hypothesis, that dogs have relatively stable colon microbial communities and episodes of diarrhea lead to instability which is compounded by antimicrobial treatments for specific pathogens, can be addressed using these methods particularly if environmental exposures are limited. This work demonstrates that dogs can be used to study changes in microbial communities associated with naturally occurring diarrheas.

## 2. MATERIALS AND METHODS

### 2.1. Enrollment of study dogs and experimental design

We established a standard operating procedure for sample collection and processing that was approved by the Michigan State University Institutional Animal Care and Use Committee (2-16-2006). The study also followed all guidelines and standard protocols of the Michigan State University (MSU) Veterinary Teaching Hospital (VTH). Information on all animals included in the study is given in Table 1. A panel of eight household pet dogs was enrolled in the study over one year. These eight dogs included two pets from a single household (Pets 1 and 2 in Table 1; Pet 1 suffered one episode of diarrhea), a single pet from a second household (Pet 3 in Table 1; this pet suffered two episodes of diarrhea which were treated at the MSU VTH), a single healthy pet from a third household (Pet 4 in Table 1), two healthy pets from a fourth household (Pets 5 and 6 in Table 1), and two pets from different households presenting to the MSU VTH with diarrhea (Pets 7 and 8 in Table 1). Five control dogs from an extended genetically related family were enrolled that were housed in an MSU-closed research colony, were fed the same food daily, and were exercised indoors to prevent infections. Feces were collected from these dogs to assess repeatability and technical quality of our protocols and to provide comparisons to diarrhea cases.

All diagnostic tests (except for an ELISA test for *Giardia*) and T-RFLP analyses were conducted on DNA isolated from fecal samples. DNA of sufficient quality for T-RFLP analysis could not be isolated from one of two samples from Pet 4 or from Pets 5, 6, 7, or 8. Diagnostic PCR results are presented for all thirteen dogs: T-RFLP results are presented for the five research colony dogs and four household pet dogs (Pets 1, 2, 3, and 4). For T-RFLP analysis of fecal microbiota over time, we collected repeated fecal samples from three household pet dogs to assess variability of the microbiota based on housing and feeding regimes. As mentioned above, two of these three dogs experienced diarrhea, one of them twice. Therefore, we collected samples from these dogs during and after treatment, if any, with the prescribed antibiotic.

### 2.2. History and clinical examination

**Figure 1:**
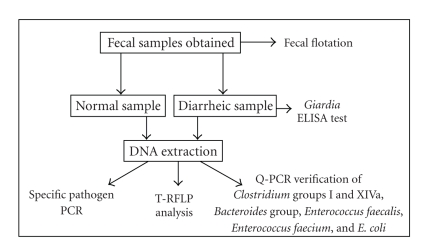
Scheme for sample processing.

The overall strategy for sampling of dogs is shown in Figure 1. The case definition for a dog with diarrhea was a dog that presented with acute diarrhea. If dogs presented to the MSU VTH with diarrhea, we instituted resampling three to five days after completion of treatment. For one of the two diarrheic household pets studied (Pet 3 in Table 1), two original samples were taken before antibiotic treatment and further samples were taken during and after antibiotic treatment; for Pet 1 in Table 1, the initial sample was diarrheic and subsequent samples were taken after resolution of disease, which was not treated.

With cases presenting with signs of acute large bowel diarrhea, the typical MSU small animal clinic protocol includes a fecal flotation, *Giardia* test, and fecal cytology. Treatment usually comprises a short course of metronidazole (10–50 mg/kg BID) and institution of a low-residue diet. In this study, our protocol involved taking a history, performing a physical examination, and initiating diagnostic tests according to standard methods currently employed in the MSU VTH.

### 2.3. Sample handling and DNA isolation

Preliminary studies were conducted to define the best method of handling fecal samples to optimize T-RFLP analysis. Using clean gloves for each animal, samples from research colony dogs and Pets 1 and 2 were taken as free catch or rectal samples; samples from other dogs were taken from the ground immediately after defecation taking care to avoid taking any part of the sample that touched the ground. Subsamples were taken from the interior of the fecal mass for analysis. The feces was placed into tryptose soy broth with 15% glycerol, mixed well, aliquoted into at least 4-5 identical subsamples; three were frozen back at −80 and one had DNA extracted that day. Samples were subjected to one of the following treatments: holding on ice only long enough for transport to the laboratory, holding at room temperature for 24 hours prior to DNA extraction, holding on ice for 24 hours prior to DNA extraction, refrigeration for 24 hours prior to DNA extraction, and freezing for 24 hours prior to DNA extraction. These conditions were intended to mimic the possible fates of clinical specimens prior to submission to the laboratory. DNA could be recovered in quantities sufficient for diagnostic PCR and T-RFLP analysis from samples subjected to all treatments, although yields were greater when samples were held on ice only long enough for transport to the laboratory. Freezing was preferred when samples could not be processed immediately in order to avoid changes in microbiota that have been documented in samples held at room or refrigeration temperatures [36–38].

Bacterial populations were recovered from 200 mg of feces by suspending samples in 300 mM sucrose solution followed by two low-speed centrifugations as described [39]. Bacteria were then collected by high-speed centrifugation and the pellets resuspended in 200 microliters 10 mM Tris 1 mM EDTA, pH 8.0. Klijn et al. [39] reported that this method results in the recovery of over 80% of aerobic/facultatively anaerobic bacteria able to grow on Columbia blood agar medium (they did not assay obligately anaerobic bacteria). Similar differential centrifugation methods have been used to isolate bacterial DNA for microbial community analysis from digesta from chicken gastrointestinal tracts [40, 41], rat cecal digesta [42], and human feces [43]. Community DNA was isolated from the harvested bacterial cells using QIAgen DNeasy Tissue Kit (QIAgen, Valencia, Calif, USA) according to the manufacturer's instructions for Gram-positive bacteria, including digestion first with 20 mg/mL lysozyme for one hour at 37°C followed by proteinase K (20 mg/mL) overnight at 55°C; DNA was then purified from the lysates using QIAgen spin columns.

### 2.4. Screening for common pathogens in normal and diarrheic dogs

Fecal samples were screened for parasites by the Cornell-Wisconsin saturated sucrose flotation technique [44]. *Giardia* tests were performed using the ProSpect Giardia-ELISA-microplate assay (Remel, Lenexa, Kan, USA) according to the manufacturer's instructions. DNA preparations were screened for the presence of DNA from the following bacterial pathogens by standard polymerase chain reaction assays using published primer pairs and cycling conditions: *Clostridium perfringens* [45], *Campylobacter* spp. [5], *Enterococcus* spp. [46], *Enterococcus faecium* [47], *Helicobacter* spp. [48], *Salmonella* spp. [49], and *Brachyspira (Serpulina)* spp. [50]. Purified DNA samples from cultured known bacterial species were used as positive controls for the PCR assays. In addition, quantitative PCR assays (Q-PCR) were performed using primers and cycling conditions developed by Rinttilä et al. [51] for *Bacteroides* spp. and related organisms, *Clostridium* group I, and *Clostridium* group XIVa. However, Q-PCR assays for *Escherichia coli* were performed as published [52]; Q-PCR assays for *Enterococcus faecium*, and *Enterococcus faecalis* were performed as described below [53].

### 2.5. T-RFLP analysis

Terminal restriction fragment polymorphism analysis was conducted using the 516f and 1510r primers, PCR reaction mixture, PCR cycling conditions, and restriction enzyme digestion conditions described by Nagashima et al. [31], except that reactions were carried out using 50 ng template DNA in a total volume of 100 *μ*L. The forward primer carried a 6-FAM fluorescent probe. The PCR products were purified using QIAquick PCR purification columns (QIAgen; Valencia, Calif, USA) according to the manufacture's protocol prior to digestion with *Bsl*I (New England Biolabs, Inc., Ipswich, Mass, USA); the resulting fragments were separated by electrophoresis on an automated DNA sequencer (ABI Prism 3100) at the MSU Genomic Technology Support Facility. An internal lane standard (MapMarker1000; BioVentures, Murfreesboro, Tenn, USA) was added to every sample, and the standard peak sizes were used by the GeneScan Analysis software to compute peak sizes. Electropherograms were stored as computer files for later analysis.

### 2.6. T-RFLP data analysis

Analysis of T-RFLP *Bsl*I peak patterns was conducted as follows. Only peaks corresponding to DNA fragment lengths between 100 and 990 bp in length, having a height of at least 25 fluorescent units, and contributing at least 1% of the total area under the electropherogram were considered; electropherograms having a total area less than 5000 fluorescence units were not analyzed. Likely identities of the phylogenetic groups of bacteria detected were determined manually by comparing peak fragment sizes to fragment sizes assigned to various bacterial groups by Nagashima et al. [31], allowing for an error of ±1 bp. Peaks that did not fall into the size classes defined by Nagashima et al. [31] were combined to form an “unknown” class. The identities of the bacterial groups and the range of peak sizes that contributed to each group are shown in Table 2. The community profile consisted of a list of the bacterial groups present or absent in a sample and the % area under the electropherogram contributed by each group.

For cluster analysis, the % area data were fourth-root transformed, and single linkage cluster analysis using the Bray-Curtis similarity index was performed on the transformed data [54]; a dendrogram was constructed, and its stability was evaluated using the jackknife procedure [55]. These calculations were performed using utilities made available online by John Brzustowski at http://www2.biology.ualberta.ca/jbrzusto/cluster.php. The Bray-Curtis similarity index is widely used in community ecology studies because it is less affected than other indices by differences involving rare, low abundance organisms and it is thought to perform better with datasets containing widely differing sets of communities [55, 56]. This index takes into account both the presence and absence of peaks and differences in the areas under same-sized peaks in the electropherograms, which indicate differences in the proportions of a particular organism in two populations.

We also calculated the descriptive microbial community parameters developed specifically for molecular ecological fingerprinting by Marzorati et al. [57]; the community parameters summarize community richness (*R*_0_), functional organization (*Fo*, evenness), and dynamics (% change and Δ_*t*_). These parameters were calculated and interpreted for T-RFLP profiles generated in this study as described in Marzorati et al. [57], except that the Bray-Curtis similarity index was used in the calculation of the % change parameter instead of the similarity index used by Marzorati et al. [57]. Finally, patterns of variability in T-RFLP profiles for Pet 3 over time were analyzed by the regression method of Collins et al. [58]. Euclidean distances between microbial communities at different time points were calculated using utilities made available online by John Brzustowski at http://www2.biology.ualberta.ca/jbrzusto/cluster.php; regression analysis was performed using SigmaStat 3.1 (Systat Software, Point Richmond, Calif, USA).

### 2.7. Quantitative real-time PCR method and analyses

DNA extracted from feces (as previously described) was used as the template in species-specific Q-PCR assays. *Bacteroides/Prevotella/Porphyromonas* and *Clostridium* groups I and XIVa assays were performed using the primer sequences from Rinttilä et al. [51]. *Escherichia coli* Q-PCR assays were performed using primer sequences from Khan et al. [52]; *Enterococcus faecalis* and *Enterococcus faecium* Q-PCR assays were performed using primer sequences from Firmesse et al. [53]. In all assays, 25 *μ*L reactions were performed in triplicate for each sample with iQ SYBR Green Supermix (Bio-Rad, Hercules, Calif, USA) and 250 ng of fecal DNA. The following cycling protocol was used for *Clostridium* group I, *Clostridium* group XIVa, *Bacteroides*, and *E. coli*: 95°C for 3 minutes and 40 repeats of 95°C for 10 seconds, specific annealing temperature for 30 seconds. Cycling parameters for *E. faecium* were 95°C, 3 minutes; 40 cycles of 63°C for 10 seconds. Cycling parameters for *E. faecalis* were 95°C, 3 minutes; 40 cycles of 64.5°C for 10 seconds. Each species-specific assay was optimized for primer concentration and annealing temperature. *Bacteroides* species assay used 6.25 pm of each primer per reaction with an annealing temperature of 65°C. *E. coli* assays used 6.25 pm of each primer per reaction and an annealing temperature of 63.3°C. *Clostridium* Groups I and XIVa used 7.5 pm of each primer per reaction with annealing temperatures of 58.9°C and 57°C, respectively. All Q-PCR assays included a 6-point standard curve in triplicate (*R* ^2^ > 0.90) and three no-template controls containing all other reaction components on a Bio-Rad iQ5 Real-Time PCR Detection System (Bio-Rad, Hercules, Calif, USA). Three DNA preparations were purchased for the standard curves: *Clostridium perfringens* for *Clostridium* group I (Sigma Aldrich, St. Louis, Mo, USA), *Ruminococcus productus* VPI 4299 for *Clostridium* group XIVa, and *Bacteroides fragilis* VPI 2553 (DNA purchased from American Type Culture Collection, Manassas, Va, USA). Three DNA preparations for the standard curves were prepared in our laboratory by the CTAB method of Ausubel et al. [59] from strains *E. coli* DH5-*α*, *Enterococcus faecalis* 19433, and *Enterococcus faecium* 19434.

Bio-Rad iQ5 PCR detection system software (Bio-Rad, Hercules, Calif, USA) was used to calculate the Ct value for each reaction, the mean Ct value for each set of triplicates, and the amount of target DNA using values derived from the standard curves. The statistic PV, which quantifies variability in population abundance over time, was calculated for each of the organisms assayed using the Q-PCR data [60].

## 3. RESULTS

### 3.1. Dogs enrolled in study

Information on sex, age, breed, and diet of all dogs enrolled in the study is given in Table 1. Five control dogs were identified that lived in MSU colonies, were fed the same food daily, and were exercised indoors. These dogs were from a family developed to study an inherited disorder unrelated to the GI tract. As such, dogs were full or half siblings, or their parents. We also collected feces from four normal dogs living in households to assess variability of the microbiota based on housing and feeding regimes. Of the household pets, two were supervised outside (e.g., walked on leash), one was allowed to roam freely in a suburban environment, and one was allowed to roam freely in a rural environment. In addition, two of the four dogs living in households experienced diarrhea, one of them twice, after collection of the normal samples. We, therefore, collected samples from these dogs during and after treatment, if any, with the prescribed antibiotic.

Fecal samples were screened for *Giardia* by ELISA. One of two dogs (Pet 8 in Table 1) presenting to the MSU Small Animal Clinic with diarrhea was positive for *Giardia*; all others were negative. Fecal samples were screened for other parasites by fecal flotation; all were negative. DNA was extracted from each fecal sample and characterized using PCR for seven known enteric pathogens. Two of the five research colony dogs carried *Enterococcus faecium, Campylobacter* spp., and *Helicobacter* spp. (Research dogs 1 and 5). Two others of the five carried *Enterococcus* spp. and, * Helicobacter* spp. (Research dogs 2 and 4). The fifth research colony dog carried only *Enterococcus faecium* (Research dog 3). Diagnostic PCR assays performed on fecal DNA obtained from the seven *Giardia*-negative household pets showed that five (Pets 1, 2, 3, 4, and 5) had at least one sample positive for *Enterococcus* spp., *Helicobacter* spp., and *Campylobacter* spp. during the sampling period; all three organisms were detected simultaneously in many samples. Pet 6 was positive for *Campylobacter* spp. and *Enterococcus* spp. but not *Helicobacter* spp. Pet 7, which presented to the MSU Small Animal Clinic with diarrhea, was positive for both *Enterococcus* spp. and *Helicobacter* spp. All thirteen dogs were negative for *Salmonella* spp. and *Brachyspira* spp.

### 3.2. Bacterial communities in research colony dogs

**Figure 2:**
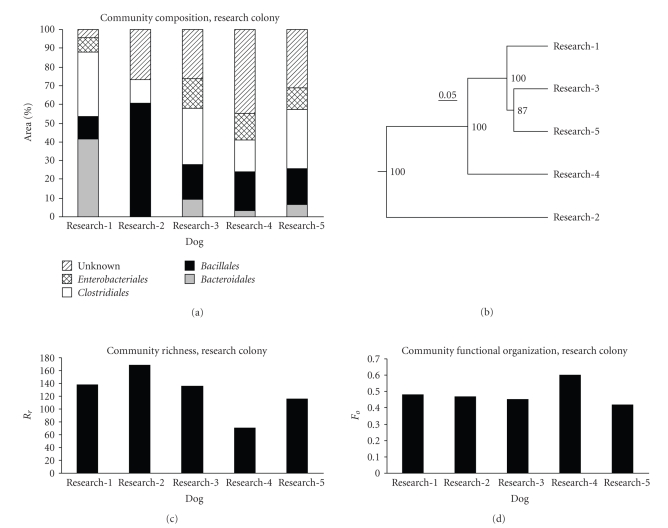
*T-RFLP analysis of fecal samples from research colony dogs*. Panel (a) community composition expressed as % area of peaks under the T-RFLP electropherogram; bacterial groups were combined at the taxonomic level of order. Panel (b) dendrogram based on Bray-Curtis similarities of community composition among the five dogs; numbers at nodes indicate percentage of trees having an equivalent node in jackknife analysis. Panel (c) community richness parameter, *Rr*. Panel (d) community functional organization, *Fo*.

Results of T-RFLP analysis of fecal samples from research colony dogs are shown in Figure 2. Community composition is shown in Panel (a); for this figure, % areas of peaks corresponding to the bacterial groups assigned by Nagashima et al. [31] were combined at the taxonomic level of order as follows: *Bifidobacteriales* (peak *Bsl*I 124), *Bacillales* (peaks *Bsl*I 322, 520, and 657), *Bacteroidales* (peaks *Bsl*I 317, 469, and 853), *Clostridiales* (peaks *Bsl*I 370, 494, 749, 919, and 955), *Enterobacteriales* (peak *Bsl*I 940), and Unknown (all peaks not classified in one of the previous groups). While a number of the *Bsl*I peaks defined by Nagashima et al. [31] contain organisms from more than one order, inspection of Table 1 in their publication shows that members of a single genus do dominate almost all peaks. The taxonomic level of order also captures some aspects of the physiology of intestinal bacteria that may be important in determining colonic health.

The fecal bacterial community composition of the genetically related research colony dogs was similar in four of five dogs (Figure 2, panels (a) and (b)). Dogs 1, 3, and 5 are housed in the same room and are the three research animals that were PCR-positive for *Enterococcus faecium*. The values of the community richness measure *Rr* for these communities are shown in Figure 2, Panel (c). These values were calculated using all peaks in each profile, not the combined order level data shown in Panel (a); values were comparable to those reported by Marzorati et al. [57] for microbially rich environments such as soils and sediments. Values of the community functional organization parameter (*Fo* reflects the “evenness” of the pattern of the relative abundances of the organisms) are shown in Figure 2, Panel (d); values ranged from 0.42 to 0.60. Marzorati et al. [57] have characterized *Rr* values >30 as indicating environments capable of sustaining large, diverse communities. *Fo* values for the research colony dog fecal communities fall around 0.45, a value which Marzorati et al. [57] have characterized as indicating a balanced community able to continue functioning properly during perturbations.

### 3.3. Household pet dogs

**Figure 3:**
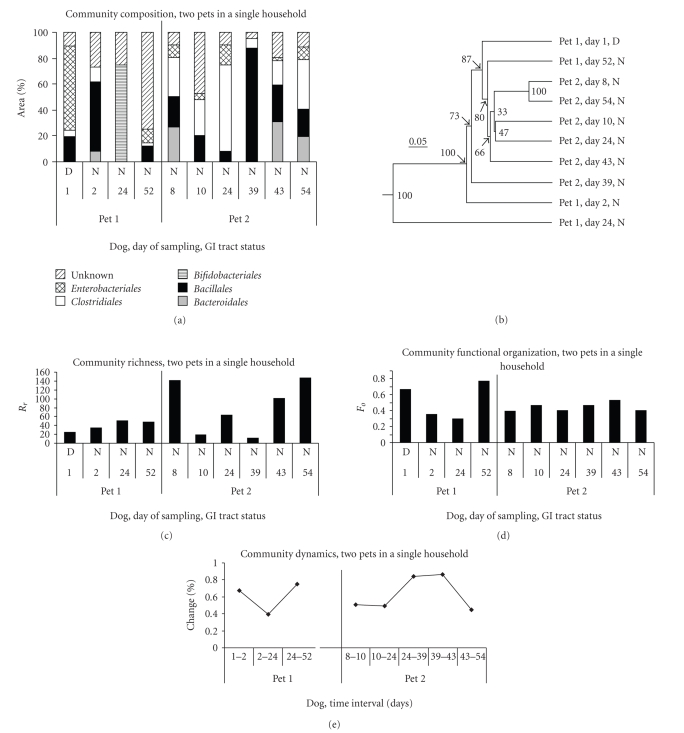
*T-RFLP analysis of fecal samples from two pets in a single household*. Panel (a) community composition expressed as % area of peaks under the T-RFLP electropherogram; bacterial groups were combined at the taxonomic level of order. Panel (b) dendrogram based on Bray-Curtis similarities of community composition among the samples from the two dogs; numbers at nodes indicate percentage of trees having an equivalent node in jackknife analysis. Panel (c) community richness parameter, *Rr*. Panel (d) community functional organization, *Fo*. Panel (e) % change parameter for community dynamics.

Results of T-RFLP analysis of fecal communities from two pet dogs (Pets 1 and 2) living in the same household and followed over time are shown in Figure 3. These dogs were genetically different and were fed different dry diets: an adult diet and a puppy diet from the same manufacturer. The adult dog (Pet 1) was allowed to roam freely in a rural environment, while the puppy (Pet 2) was exercised outdoors under the owners' supervision. The episode of diarrhea in Pet 1 on day 1 was due to dietary indiscretion and resolved by the next day; it was characterized by a “bloom” of *Bsl*I 940 (*E. coli*). Community compositions of fecal samples from the two dogs were dissimilar and exhibited considerable variation over time (Figure 3, Panel (a)). Nevertheless, five of six community composition patterns for Pet 2 did cluster together in the dendrogram shown in Figure 3, Panel (b). Values of the richness parameter *Rr* for Pet 1 ranged from 25 to 50; values of *Rr* for Pet 2 ranged from 11 to 147 (Figure 3, Panel (c)). These *Rr* values were generally lower than those obtained for the research colony dogs. *Fo* values ranged from 0.30 to 0.77 for Pet 1 and from 0.39 to 0.53 for Pet 2 (Figure 3, Panel (d)). *Fo* values for both dogs were more constant than either community composition or *Rr* values. The community dynamics % change parameter was calculated for sequential samples (Figure 3, Panel (e)); because the time intervals between samples were not equal, we did not calculate the rate of change parameter Δ_*t*_. Values for Pet 1 ranged from 0.40 to 0.75 and for Pet 2 from 0.45 to 0.86. These % change values are much higher than those reported for other kinds of microbial communities by Marzorati et al. [57].

One T-RFLP profile was obtained from another household pet (Pet 4); this animal was fed a commercial adult diet and occasional table scraps and allowed to roam freely in a suburban environment; both *Rr* and *Fo* values were low: 6.0 and 0.31, respectively.

### 3.4. Household pet dog with repeated diarrhea

**Figure 4:**
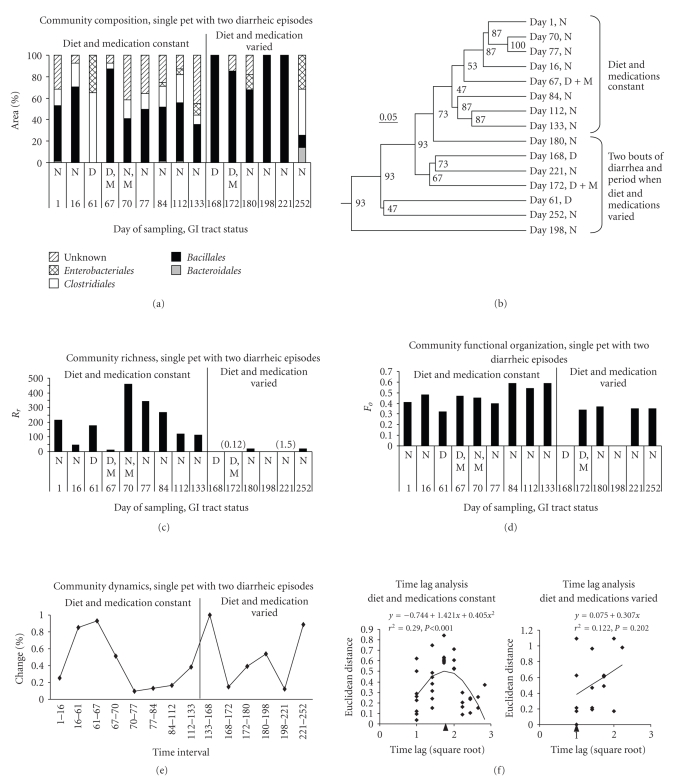
*T-RFLP analysis of fecal samples from a single pet with two diarrheic episodes*. Panel (a) community composition expressed as % area of peaks under the T-RFLP electropherogram; bacterial groups were combined at the taxonomic level of order. Panel (b) dendrogram based on Bray-Curtis similarities of community composition among the samples from this dog; numbers at nodes indicate percentage of trees having an equivalent node in jackknife analysis. Panel (c) community richness parameter, *Rr*. However, *Rr* is not reported when there was only a single peak ≥1% of the area under the electropherogram (days 168 and 198); the value of *Rr* was 0.12 on day 172 and 1.5 on day 221. Panel (d) community functional organization, *Fo*. However, *Fo* is not reported when there was only a single peak ≥1% of the area under the electropherogram (days 168 and 198). Panel (e) % change parameter for community dynamics. Panel (f) time lag analysis. Arrows below the *X*-axis indicate the day of onset of an episode of diarrhea.

Results of T-RFLP analysis of fecal samples from a household pet dog (Pet 3) experiencing two separate episodes of diarrhea are shown in Figure 4. This animal was fed a commercial weight control dry diet two to four times higher in fiber than the diets of the other dogs in the study, and was consistently supervised by the owner when outdoors in a suburban environment. At the time of enrollment in the study, this pet was being treated for osteoarthritis with carprofen at 1.0 mg/kg BID; on day 121 after enrollment, the dosage was increased to 2.0 mg/kg BID. Moderate anorexia developed on day 151, and the owner began supplementing the diet in various ways to stimulate appetite; this supplementation continued throughout the rest of the study. After the second episode of diarrhea on day 168, treatment with carprofen was discontinued and treatment with tramadol (2.7 mg/kg TID) was instituted. Tramadol was discontinued due to vomiting on day 219 and deracoxib (1.4 mg/kg SID) was initiated; this drug was also not well tolerated and was discontinued on day 233. No further analgesics were given during the study.

Results of pathogen-specific PCR assays for this animal are shown in Table 3. *Campylobacter* and *Helicobacter* spp. were detected by PCR assay in 11 of 15 samples; the *Campylobacter* spp. PCR assay was positive in two samples taken on the day of onset of the first episode of diarrhea. *Enterococcus faecalis* and *E. faecium* assays were positive following the onset of the first episode of diarrhea. *E. faecalis* was not detected subsequently by this assay, while *E. faecium* levels were below the limit of detection until the onset of the second episode of diarrhea when the dog became positive over a course of 30 days.

Community composition was similar in the normal samples taken on days 1, 17, 70, 78, 85, 113, and 134. Episodes of diarrhea occurred beginning on days 61 and 168; both episodes were treated with metronidazole (1st treatment starting on day 65 (14.0 mg/kg BID, for 10 days) and 2nd treatment starting on day 172 (14.0 mg/kg BID for 6 days)), and a brief period on a low-residue diet. Because the second episode of diarrhea occurred 107 days after the first and the microbiota returned to its starting composition during the interval, the second episode was probably not an antibiotic-associated diarrhea. Community composition returned to its starting composition after the first episode of diarrhea but not after the second; the two episodes were also different in character, the first being dominated by *Enterobacteriales* and *Clostridiales* and the second by *Bacillales*. Simple fecal cytology performed by MSU VTH staff at the onset of the second episode of diarrhea was read out as bacterial overgrowth. The community did not begin to return to its previous composition for an extended period after the second episode of diarrhea. On the last day of sampling, representatives of all the orders previously present were detected, but their proportions were altered from those in the normal samples. The dendrogram showing the similarities of the communities at different times (Figure 4, Panel (b)) also demonstrates this pattern: most of the samples taken prior to day 168 cluster together and away from the diarrheic samples and the normal samples taken after the second episode of diarrhea.

Changes in community richness parameter *Rr* values are shown in Figure 4, Panel (c). Community richness dropped to a value of 11.2 following institution of metronidazole treatment for the first episode of diarrhea but rebounded to a high level (460) and then declined slowly over the next month. Richness was severely reduced during the second episode of diarrhea and did not return to even moderate levels; repeated changes in medications and diet were made during the period after the second episode of diarrhea. Changes in *Fo* values (Figure 4, Panel (d)) were less pronounced but consistently somewhat lower after the second episode of diarrhea.

Changes in the community dynamics parameter % change also reflect the events described above (Figure 4, Panel (e)). Large values (0.85 to 0.99) are associated with the two episodes of diarrhea. Prior to the first episode of diarrhea and after recovery from it, the % change values ranged from 0.09 to 0.38 and were comparable to those reported for other kinds of communities by Marzorati et al. [57]. Marzorati et al. characterized % change values around 0.10 as indicating that the community is stable; new organisms are able to become established but do not interfere with community function. Very high % change values were associated with the diarrheic episodes in Pet 3, and after the second episode, the % change values continued to fluctuate, indicating that the community did not return to stability. This period of instability coincided with the period of repeated changes in medications and diet.

Temporal variability in the T-RFLP data was also analyzed using the regression method of Collins et al. [58] (Figure 4, panel (f)); the period when diet and medication were constant and the period when both varied were analyzed separately. The period when diet and medications were constant, which included the first episode of diarrhea, exhibited a phase with a positive slope followed by a phase with a negative slope. This pattern can be interpreted as showing a directional change in community composition after a perturbation followed by a return to the starting condition [58]. The period when diet and medications were varied did not yield a significant regression; according to Collins et al. [58], this result indicates fluctuating changes in community composition over time.

### 3.5. Q-PCR analysis of fecal samples from a household pet dog experiencing repeated episodes of diarrhea

**Figure 5:**
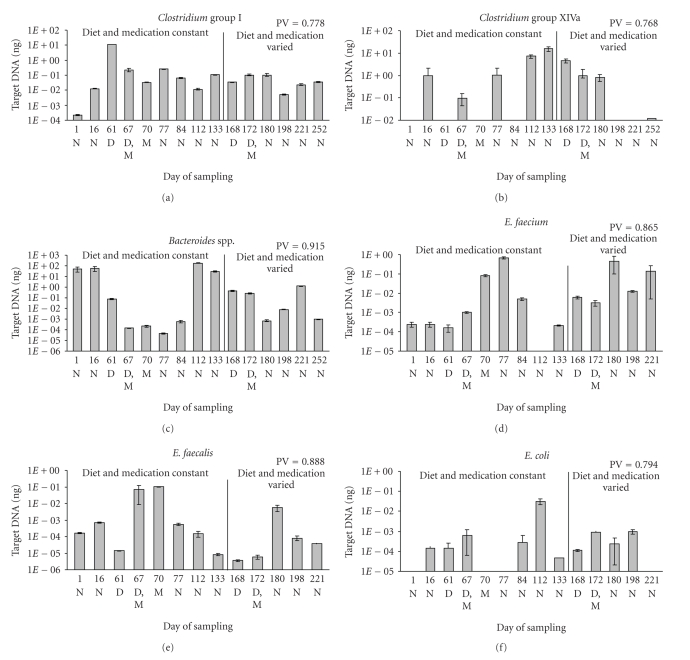
*Q-PCR analysis of bacterial groups in fecal samples from a single pet with two diarrheic episodes*. Panel (a) *Clostridium* group I; Panel (b) *Clostridium* group XIVa; Panel (c) *Bacteroides* spp.; Panel (d) *E. coli*; Panel (e) *E. faecium*; Panel (f) *E. faecalis*. *R* ^2^ values for the standard curves were 0.995, 0.986, 0.956, 0.958, 0.980, and 0.998, respectively. Error bars indicate the standard deviation of triplicate samples. Lack of a bar indicates that no product was detected in 40 cycles. The sample for day 252 was exhausted before Q-PCR for *E. faecium, E. faecalis,* and *E. coli* was performed. Population variability (PV) values are shown at the upper right of each panel.

To explore further the results for Pet 3 obtained from T-RFLP analysis, we performed quantitative real-time PCR on the same DNA samples used for T-RFLP. Q-PCR results are shown in Figure 5, Panels (a) to (f). Population variability (PV) values were high (range: 0.768–0.915) for all organisms assayed. *Clostridium* groups I and XIVa were analyzed separately; group I contains the potential enteric pathogen *C. perfringens*. (*C. difficile* is a member of *Clostridium* group XI and would not be detected by these assays.) Both groups were detected by Q-PCR throughout the sampling period. *Clostridium* group I levels were relatively constant, except for an 880-fold increase coincident with the first episode of diarrhea (day 61); the *Clostridiales* peaks comprised 55% of the total area under the electropherogram for that sample in the T-RFLP analysis (Figure 4(a)), and the *Bsl*I 749 peak comprised 40% of the total area under the electropherogram (data not shown). The *Bsl*I 749 peak is included in the *Clostridiales* portion of the bar in Figure 4(a). The expected *Bsl*I peak size for *Clostridium perfringens* is approximately 750 bp. Once this result was obtained, we performed a specific PCR assay for *Clostridium perfringens* [45]. That assay was negative prior to the first episode of diarrhea, it became strongly positive in the first diarrheic sample (Table 3), and remained so in all but one sample for about three weeks. Four weeks after the last positive sample, the assay was negative and remained so until the onset of the second episode of diarrhea, when it became strongly positive again. It remained positive throughout the second metronidazole treatment and was still positive twelve weeks later.

*Clostridium* group XIVa levels were more variable and did not exhibit any pattern with respect to either episode of diarrhea. Levels of *E. coli* exhibited a peak on day 112 but also did not exhibit any pattern with respect to either episode of diarrhea.

Levels of *E. faecium* and *E. faecalis* both rose after the initiation of metronidazole treatment during both episodes of diarrhea. *E. faecium* rose 4000-fold between the onset of the first episode of diarrhea (day 61) and day 77 and 77-fold between the onset of the second episode of diarrhea (day 168) and day 180. *E. faecalis* rose 7000-fold between the onset of the first episode of diarrhea (day 61) and day 70 and 1500-fold between the onset of the second episode of diarrhea (day 168) and day 180.

Q-PCR assay detected *Bacteroides* in all samples. Levels of *Bacteroides* spp. were highest in normal samples taken during the period when diet and medications were constant. *Bacteroides* spp. decreased approximately 10^5^-fold during and following the first episode of diarrhea but returned to the original levels. Levels decreased approximately 10^4^-fold during the second episode of diarrhea and fluctuated thereafter. This result is in marked contrast to the T-RFLP assay, which seldom detected *Bacteroides* at levels exceeding 1% of the total area under the electropherogram.

## 4. DISCUSSION

Screening for specific pathogens by PCR revealed that most animals carried one or more potential enteric pathogens in their fecal microbiota even when they had no clinical signs. Thus, many cases of “spontaneous” diarrhea in dogs—and by extension, sporadic diarrhea in humans—may be caused by organisms already present in the GI tract following perturbation of the microbiota by an environmental factor rather than by a pathogen acquired from another source [6, 61]. The hypothesis that alterations in the microbiota may inhibit or facilitate disease processes has been invoked in the context of chronic inflammatory bowel diseases; our results support the idea that there may be a significant role for the microbiota in acute infectious disease processes. Other clinical and experimental studies suggest that the relative balance of aggressive and protective bacterial species is altered in inflammatory diseases such as Crohn's disease (CD), ulcerative colitis (UC), and pouchitis. In a review of current work in this area, it was postulated that overly aggressive immune responses to a subset of commensal (nonpathogenic) enteric bacteria in genetically predisposed individuals result in disease [62]. Recently, Frank et al. showed that CD patients, UC patients, and noninflamed controls had statistically significantly different microbiotas based on culture-independent rRNA sequence analysis of cloned libraries [63]. Because it was based on surgical samples of colon, this study provided a survey of gut-wall associated microbiota relevant to Inflammatory Bowel Disease. Here, as in all studies using methods based on SSU rRNA, bacterial “numbers” were recognized to be relative estimates reflecting gene copy numbers and not indicative of causation. However, microbiota surveys can provide candidates for hypothesis testing of causation. In our study, fecal samples could be argued to best represent “diagnostic” samples that would be taken from a host presenting with acute diarrhea. It is acknowledged that further work will be needed to document the ability of readily available fecal samples to represent different locations within the colon to document the etiologies of these diarrheas [64].

The influence of diet and medication on the intestinal microbiota has been studied directly in animals and humans [17, 18, 21, 29, 62]. Although it was not our intent to study such effects, our results confirm that the composition of the intestinal microbiota is quite sensitive to changes in environmental factors such as changes in diet and/or medications as well as exposure to the microbiotas of other animals. The relatively consistent microbiotas of the research colony dogs are in marked contrast to the fluctuations observed in Pet 1, which roamed freely in a rural environment. The microbiotas of Pets 2 and 3, which were more closely supervised when out of doors, showed less variability than that observed in Pet 1 but more than in the research colony dogs. Finally, fluctuations in the microbiota of Pet 3 became much more pronounced when diet and medication were changed.

Comparison of results from T-RFLP and Q-PCR results for Pet 3 showed that both methods can detect variation in the microbiota associated with events such as diarrheal episodes and changes in diet and medication. The extensive variability in the abundances of the different groups of organisms evident in the T-RFLP data was also evident in the Q-PCR results for individual groups and organisms; population variability (PV) values for all organisms assayed by Q-PCR were high. This result is not surprising given the wide range of target DNA concentrations detected by this assay (lowest detected level, 3.6 × 10^−6^ ng for *E. faecalis*; highest detected level, 1.8 × 10^2^ ng for *Bacteroides*).

The T-RFLP analysis indicated that there was a large increase in *Clostridiales,* including the *Bsl*I 749 bp peak defined by Nagashima et al. [31], on day 61, the day of onset of the first episode of diarrhea. Q-PCR analysis indicated an increase in *Clostridium* group I, of which *C. perfringens* is a member, which coincided with the onset of the first episode of diarrhea. Diagnostic PCR assays for *C. perfringens* showed that this organism was temporally associated with the onset of both episodes of diarrhea. But because the time intervals that elapsed between samples were long, we cannot determine whether the increases in *Clostridium* group I or the detection of *C. perfringens* reflected causes or consequences of the episodes of diarrhea. The data thus suggest but do not prove that the illness was caused by a member of *Clostridium* group I, possibly *C. perfringens*. However, *C. perfringens* remained at detectable levels during the period when diet and medications varied. This result suggests that the instability of the microbial community during this period facilitated the growth of this potential pathogen.

T-RFLP analysis also indicated large increases in *Bacillales;* which includes lactobacilli, streptococci, and enterococci; subsequent to metronidazole treatment. In Q-PCR analysis, both *E. faecium* and *E. faecalis* exhibited repeated substantial increases after the initiation of metronidazole treatment, and after the second episode of diarrhea, *E. faecium* levels did not return to those seen at the beginning of the study. These results suggest that, in view of the potential of both *E. faecium* and *E. faecalis* to have deleterious effects on the GI tract, the use of metronidazole as a first-line treatment for canine diarrhea should be re-evaluated.

Perturbations in *Bacteroides* spp. levels associated with the two episodes of diarrhea were also apparent; levels of these organisms decreased during both episodes of diarrhea and became unstable during the period when diet and medication were varied. Since *Bacteroides* spp. are major components of the colon microbial community and essential to its function, such fluctuations might be expected to have repercussions for colon health.

The discrepancy between the two methods in detecting the genus *Bacteroides* in this animal would be most simply explained as a relative inability of the universal eubacterial 16S primers used to bind to the 16S rDNA sequences of the particular *Bacteroides* spp. predominating in that dog compared to the more specific primers used in the Q-PCR assay. However, members of the *Bacteroidales* were detected by the primers used in T-RFLP analysis in four of the five research colony dogs and in Pets 1 and 2. BLAST searches indicated that the region of homology between the Nagashima et al. [31] reverse primer to the 16S rDNA sequence of (1) some but not all strains of *Bacteroides eggerthii*, (2) some but not all strains of *B. stercoris*, and (3) some but not all strains of *B. caccae* consists only of the seven bases from nucleotide 11 to nucleotide 17 of the 19-base primer. This limited homology was even present in the 16S rDNA sequences of the type strains of *B. stercoris* and *B. caccae*. No PCR product would be obtained from DNA of these strains. Also, BLAST searches indicated that the 16S primers used for the Q-PCR studies [51] would be expected to amplify rDNA of all strains of these three species. However, *B. eggerthii, B. stercoris*, and *B. caccae* are found in humans; in a cloned library study, sequences closely related to *B. stercoris* were obtained from dogs [20]. Of the *Bacteroides* spp. sequences obtained from dogs in the latter study, one was very similar to that of *B. stercoris*, three were similar to that of *B. vulgatus,* and four were not similar to those of any of the published *Bacteroides* spp. used in the analysis. The same study also indicated that the *Bacteroides* spp. sequences from the fecal microbial communities of humans, dogs, cats, and gulls clustered together and separately from those of cattle and elk.

We applied the community characterization schema proposed by Marzorati et al. [57] to the dog fecal bacterial communities studied here and related them to what we know about the management and clinical presentation of the dogs. The research colony dog communities were characterized by high richness and intermediate “balanced” levels of functional organization. Because of the constancy of the environment of these dogs, we predict that their communities would experience low-to-medium dynamics similar to those observed in normal samples from Pet 3. The communities of Pets 1, 2, and 4 were generally characterized by lower richness, balanced functional organization, and high levels of dynamic change for Pets 1 and 2.

The normal communities of Pet 3 prior to and following the first episode of diarrhea were characterized by high richness, medium levels of dynamic change, and balanced functional organization, while the diarrheic samples were characterized by low richness, high levels of dynamic change, and low levels of functional organization. Communities in the normal samples taken following the second episode of diarrhea were characterized by continued low richness, fluctuating levels of dynamic change, and somewhat lower levels of functional organization than previous normal samples. The fluctuating levels of dynamic change were temporally correlated with changes in diet and medication. Time lag analysis indicated that samples taken when diet and medication were constant—the initial samples, samples taken during the first episode of diarrhea, and samples taken prior to the second episode of diarrhea—showed a recognizable pattern of disturbance followed by a return to the initial condition. Samples taken when diet and medications were varied—beginning with the onset of the second episode diarrhea and continuing to the end of the study—did not exhibit any directional changes but instead showed random fluctuation. The general agreement between these complementary analyses suggests that concepts from macroecology will be useful in interpreting data from microbial communities.

According to Marzorati et al. [57], the level of the community richness parameter *Rr* is indicative of the carrying capacity of the environment; diet is one obvious environmental variable that might affect the carrying capacity in the GI tract. The similar values of *Rr* obtained for research colony dogs fed and housed under controlled conditions supports this idea. However, Pet 2 was genetically related to the research colony dogs and was fed a similar diet, but exhibited lower community richness. This free-roaming dog probably had a much more variable intake than the research colony dogs, so values for apparent carrying capacity might have been influenced by the highly dynamic nature of the microbial community in this animal. A similar effect can be seen in the richness parameter values for Pet 3; *Rr* varied widely subsequent to perturbation and periods of dynamic change in the community due to episodes of diarrhea.

The level of dynamic change also varied considerably in Pets 1, 2, and 4, and was correlated with environmental factors in that Pets 1 and 2 experienced both more environmental variation and consistently higher levels of change in community composition than did Pet 3 during healthy periods. The community functional organization parameter *Fo* is held by Marzorati et al. [57] to reflect the resistance of the community structure to perturbation. If *Fo* values do predict community resiliency, then such resiliency may explain why both Pet 1 and Pet 2, which had relatively robust *Fo* values, were mainly non-diarrheic in spite of having high levels of dynamic change and substantially shifting community compositions. In the case of Pet 1, the latter phenomena may have been due to varied intake of substances from the environment; in the case of Pet 2, a puppy, these phenomena may have been due to maturation processes in the GI tract. In addition, after the second episode of diarrhea in Pet 3, *Fo* values declined and the community appeared to become less stable under the influence of changing diet and medication regimes; this observation further supports the connection between functional organization and resiliency.

Studies with larger sample sizes are required to substantiate these apparent correlations in a rigorous way. Also, more work is clearly needed to delineate the variability of the microbiota of the healthy GI tract before embarking on detailed studies of disease states. However, based on these observations, we would predict that if the normal or background variability is substantial, it may prove exceedingly difficult to detect relevant changes in heterogeneous populations such as individuals enrolled in clinical trials.

## 5. CONCLUSIONS

We hypothesized that dogs have a stable composition of the colon microbial community and that episodes of diarrhea lead to long lasting changes in community composition and/or function; furthermore, treatment for specific pathogens can compound these effects. Outbred monogastric animals like dogs can serve as easily manipulable models to address approaches for problems of the human GI tract. Thus, the microbiota of dogs was studied during diarrheic episodes and compared to those of healthy control dogs to make a preliminary assessment of the contribution of members of the microbiota to acute diarrheal disease processes. We found that (1) four of five dogs living in an environment expected to provide the least exposure to factors that might alter the GI tract microbiota had similar microbiotas, (2) the microbiotas of dogs kept in more variable environments were correspondingly more variable, (3) acute episodes of diarrhea resulted in large-scale changes in the GI tract microbiota, and (4) when the diet and medications of a dog having a previously stable microbiota were changed repeatedly, the GI tract microbiota also changed repeatedly, ultimately reducing richness. The high levels of variability we encountered in the pet dogs indicate that descriptive population-based microbiota studies may be so fraught with variation within and between individuals that meaningful patterns and changes may be hard to distinguish from the “noise.” Either longitudinal studies of individuals under relatively constant environmental regimes (Pet 3) or model-based studies of groups of individuals under strictly controlled environments (research colony dogs) with planned experimental interventions could be expected to yield interpretable results. The consistency of the microbial communities in the research colony dogs and the changes we were able to observe in Pet 3 indicate that it is possible to establish baseline starting conditions and that the methods employed in these studies can be used to detect and delineate changes in fecal microbial communities. We expect these considerations derived from this useful animal model to apply with equal force to studies in humans.

## Figures and Tables

**Table 1:** Characteristics of dogs enrolled in the study.

	Breed	Sex	Age	Diet (%)			Comments
				protein	fat	fiber	
Pet 1	Beagle × schnauzer; related to research colony dogs	F	4 years	21.5	13.0	3.0	Time series; one diarrheic samples (dietary indiscretion; no treatment), three normal samples
Pet 2	Labrador retriever puppy; same household as Pet 1	M	9 weeks	26.0	8.5	3.0	Time series; six normal samples
Pet 3	Golden retriever	M	12 years	25.0	6.0–8.0	12.0	Time series with normal and diarrheic samples (dietary indiscretion; metronidazole treatment)
Pet 4	Golden Retriever	M	6 years	22.0	12.0	5.0	One normal sample
Pet 5	Dachshund	M	7 years	22.0	13.0	3.0	Normal sample
Pet 6	American Eskimo mix	M	3 years	22.0	13.0	3.0	Normal sample
Pet 7	Retriever mix	F	2 years	ND	ND	ND	Diarrheic sample
Pet 8	Mixed breed	F	12 years	ND	ND	ND	Diarrheic sample
Research 1	Beagle × Schnauzer	F	4 years	25.0	9.0	5.0	Normal sample
Research 2	Beagle × Schnauzer	F	6 years	25.0	9.0	5.0	Normal sample
Research 3	Beagle × Schnauzer	M	4 years	25.0	9.0	5.0	Normal sample
Research 4	Beagle × Schnauzer	F	6 years	25.0	9.0	5.0	Normal sample
Research 5	Beagle × Schnauzer	F	4 years	25.0	9.0	5.0	Normal sample

**Table 2:** Assignment of T-RFLP fragment size classes to bacterial taxa based on data of Nagashima et al. [31].

Fragment class according to Nagashima et al.[31]	Fragment size range (bp)	Predominant genus	Other genera included
*Bsl*I 110	110–115	None	*Clostridium, Eubacterium, Lactobacillus, Veillonella*
*Bsl*I 124	125–128	*Bifidobacterium*	None
*Bsl*I 317	316–319	*Prevotella*	*Lactobacillus*
*Bsl*I 332	326–338	*Streptococcus*	*Bifidobacterium, Clostridium, Eubacterium, Lactobacillus, Prevotella*
*Bsl*I 370	364–378	*Clostridium*	*Bacteroides, Bifidobacterium, Eubacterium, Lactobacillus, Prevotella*
*Bsl*I 469	464–473	*Bacteroides*	*Clostridium, Eubacterium, Prevotella*
*Bsl*I 494	487–502	*Clostridium*	*Eubacterium, Ruminococcus, Streptococcus*
*Bsl*I 520	513–519	*Enterococcus*	*Clostridium, Eubacterium, Lactobacillus*
*Bsl*I 657	655–665	*Lactobacillus; Streptococcus*	*Bacteroides, Clostridium, Enterococcus, Eubacterium, Ruminococcus*
*Bsl*I 749	748–757	*Clostridium*	*Eubacterium, Fusobacterium, Ruminococcus*
*Bsl*I 853	848–854	*Bacteroides*	*Bacteroides*
*Bsl*I 919	911–921	*Ruminococcus*	*Enterococcus, Eubacterium*
*Bsl*I 940	935–941	*Escherichia*	*Clostridium, Eubacterium, Fusobacterium, Ruminococcus*
*Bsl*I 955	955–960	None	*Clostridium, Eubacterium, Ruminococcus*

**Table 3:** Detection of potential pathogens in pet dog with two diarrheic episodes by standard PCR assay*.

Day	Status**	*Enterococcus*	*Enterococcus*	*Campylobacter*	*Helicobacter*	*Clostridium*
		*faecalis*	*faecium*	spp.	spp.	*perfringens*
1	N	−	−	+	+	−
16	N	−	−	−	+	−
61	D	−	−	+	+	+
67	D, M	+	+	−	−	+
70	N, M	+	+	−	−	−
77	N	−	−	−	−	+
84	N	−	−	−	+	+
112	N	−	±	−	+	−
133	N	−	−	−	+	−
168	D	−	+	−	−	+
172	D, M	−	+	−	+	+
180	N	−	+	−	+	+
198	N	−	±	−	+	+
221	N	−	−	−	+	+
252	N	−	−	−	+	+

*“−”: negative; “+”: positive; “±”: weak positive. All fecal flotation and *Giardia* tests were negative.

**N: normal; D: diarrhea: M: metronidazole treatment.
